# *Mycobacterium leprae* and host immune transcriptomic signatures for reactional states in leprosy

**DOI:** 10.3389/fmicb.2023.1113318

**Published:** 2023-03-27

**Authors:** Madhusmita Das, Diana David, Ilse Horo, Anouk Van Hooij, Maria Tió-Coma, Annemieke Geluk, Sundeep Chaitanya Vedithi

**Affiliations:** ^1^Molecular Biology and Immunology Division, Schieffelin Institute of Health Research and Leprosy Centre, Karigiri, Vellore, Tamil Nadu, India; ^2^Department of Infectious Diseases, Leiden University Medical Centre, Leiden, Netherlands; ^3^Department of Biochemistry, University of Cambridge, Cambridge, United Kingdom

**Keywords:** whole transcriptome microarray, *Mycobacterium leprae*, type I and type II reactions, gene expression signatures, differential expression

## Abstract

**Background:**

*Mycobacterium leprae* transcriptomic and human host immune gene expression signatures that demonstrate a plausible association with type I (T1R) and type II reactions (T2R) aid in early diagnosis, prevention of nerve damage and consequent demyelinating neuropathy in leprosy. The aim of the study is to identify *M. leprae* and host-associated gene-expression signatures that are associated with reactional states in leprosy.

**Methods:**

The differentially expressed genes from the whole transcriptome of *M. leprae* were determined using genome-wide hybridization arrays with RNA extracted from skin biopsies of 20 T1R, 20 T2R and 20 non reactional controls (NR). Additionally, human immune gene-expressions were profiled using RT2-PCR profiler arrays and real-time qPCRs.

**Results:**

The RNA quality was optimal in 16 NR, 18 T1R and 19 T2R samples. Whole transcriptome expression array of these samples revealed significant upregulation of the genes that encode integral and intrinsic membrane proteins, hydrolases and oxidoreductases. In T1R lesional skin biopsy specimens, the top 10 significantly upregulated genes are ML2064, ML1271, ML1960, ML1220, ML2498, ML1996, ML2388, ML0429, ML2030 and ML0224 in comparison to NR. In T2R, genes ML2498, ML1526, ML0394, ML1960, ML2388, ML0429, ML0281, ML1847, ML1618 and ML1271 were significantly upregulated. We noted ML2664 was significantly upregulated in T1R and repressed in T2R. Conversely, we have not noted any genes upregulated in T2R and repressed in T1R. In both T1R and T2R, ML2388 was significantly upregulated. This gene encodes a probable membrane protein and epitope prediction using Bepipred-2.0 revealed a distinct B-cell epitope. Overexpression of ML2388 was noted consistently across the reaction samples. From the host immune gene expression profiles, genes for CXCL9, CXCL10, CXCL2, CD40LG, IL17A and CXCL11 were upregulated in T1R when compared to the NR. In T2R, CXCL10, CXCL11, CXCL9, CXCL2 and CD40LG were upregulated when compared to the NR group.

**Conclusion:**

A gene set signature involving bacterial genes ML2388, ML2664, and host immune genes CXCL10 and IL-17A can be transcriptomic markers for reactional states in leprosy.

## Introduction

*Mycobacterium leprae* (*M. leprae*), the causative bacillus for leprosy, continues to infect endemic populations in tropical countries, with approximately 200,000 new cases of leprosy emerging each year globally. *Mycobacterium leprae* infects the skin and the peripheral nerves causing skin lesions with loss of sensation resulting from demyelinating neuropathy as the bacilli infect the Schwann cells of the axonal myelin in the peripheral neurons (Richardus et al., 1996). Nerve damage in leprosy is mediated by *M. leprae* infection of the Schwann cells as well as exacerbated immune responses in the human host. Leprosy is manifested with a complex host immunological profile that classifies the disease into a cell-mediated immunity (CMI)—dominated tuberculoid pole (TT) and the humoral immune (HI) response—regulated lepromatous pole (LL; WHO Expert Committee on leprosy, World Health Organization, 2012). Both these poles are separated by three borderline intermediary groups that gradient from CMI towards the HI. These include the borderline-tuberculoid (BT), mid-borderline (BB) and marginal lepromatous forms (BL; Ridley and Jopling, 1966).

About 30–40% of leprosy infected individuals in the borderline forms and rarely in the polar states manifest delayed-type hypersensitivity reactions, the type 1 reaction also known as reversal reaction and the type 2 reaction known as Erythema Nodusum Leprosum (ENL; Kahawita and Lockwood, 2008; Montoya and Modlin, 2010). These inflammatory responses can occur before, during and after the treatment with multidrug therapy (MDT) and are managed by immunomodulatory drugs in high doses that often contribute to morbidity. Reactional states are a significant cause of nerve damage and associated disability in leprosy. Early detection of reactional episodes can facilitate prophylactic treatment interventions that minimize the risk of nerve damage (Kwittken, 1968; Sehgal, 1987; Nery et al., 2013).

Predictive genomic, transcriptomic and host immune biomarkers can play a critical role in detecting subclinical nerve damage and determining factors that trigger reactional states in leprosy. In leprosy endemic tropical countries, it is often challenging to characterize an individual’s immune background due to varied antigenic exposure (Yuan et al., 2021). Thus, attributing specific immune responses (cytokine and antibody quantities or human immune gene expression signatures) alone to the onset of reactional states or leprosy *per se* may offer limited applicability in developing effective diagnostics for these *M. leprae* specific immune exacerbations in leprosy (Leal-Calvo et al., 2021).

A correlative gene expression signature that originates from both *M. leprae* and human host immune system provides comprehensive predictive and prognostic information for determining the onset of these inflammatory responses (Teles et al., 2013; Manry et al., 2017; Montoya et al., 2019; Leal-Calvo et al., 2021). In this study, we conducted a cross sectional analysis to quantify relative abundance of *M. leprae* and host immune gene transcripts in localized skin lesions of leprosy cases with type I and type II reactions. A gene expression signature that demonstrates significant association with reactional states has been determined. Follow up studies are warranted to validate these expression signatures in a longitudinal cohort (Tió-Coma et al., 2021).

## Materials and methods

### Sample size

**Figure 1:**
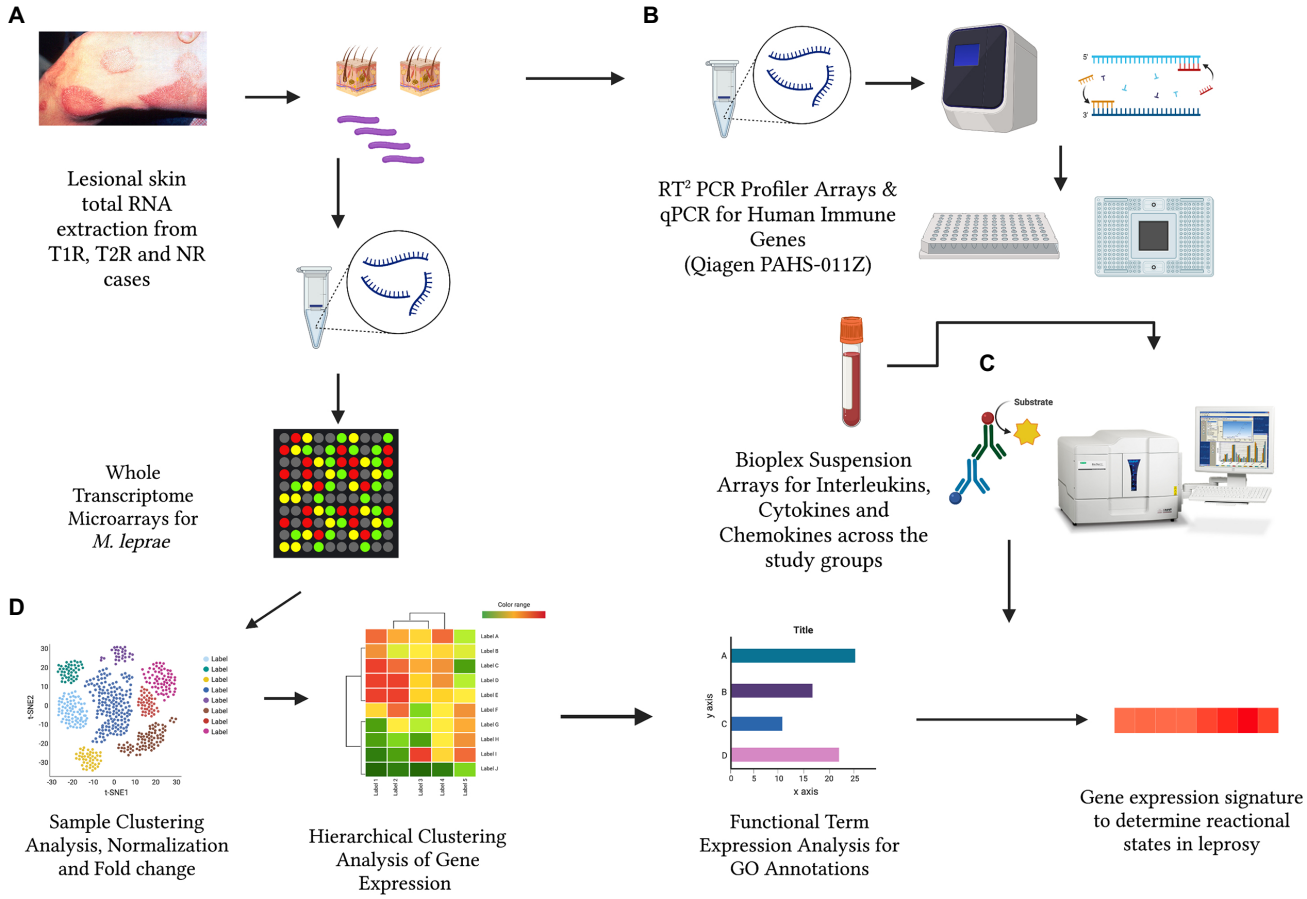
The schematic representation for the cross-sectional analysis of *Mycobacterium leprae* transcriptome **(A)**, human immune gene expression **(B)** and corresponding circulatory levels of cytokines, interleukins and chemokines **(C)** to derive a gene expression signature for reactional states of leprosy **(D)**. (Created with BioRender.com).

A total of 60 newly diagnosed untreated leprosy cases were recruited at the outpatient department of Schieffelin Institute of Health Research and Leprosy Centre in Karigiri, India. Following institutional ethical clearance, informed and written consent for participation was obtained from each subject prior to recruitment in the study following the ethical guidelines as laid down by the Indian Council of Medical Research. The sample was stratified as 20 with Type 1 Reaction, 20 with Type 2 reaction and 20 without any reaction. Post clinical examination, 5 mm x 5 mm excisional skin biopsies from skin lesions were collected of subjects for all the study groups. Clinical details of the sample were provided in Table 1. The study design and experiments were depicted in Figure 1.

**Table 1:** Clinical and demographic characteristics of the study sample.

SL NO.	Sample ID	Reactional status	Age	Gender	WHO classification	RJ classification	Bacteriological index
1	LRI 001	T2R	47	Male	MB	LL	4.75
2	LRI 002	NR	40	Male	MB	HISTOID	2.75
3	LRI 003	NR	33	Female	MB	BT	0.5
4	LRI 004	T1R	50	Male	MB	BT	0
5	LRI 005	NR	52	Male	MB	LL	3.25
6	LRI 006	T2R	48	Male	MB	LL	5
7	LRI 007	NR	15	Male	MB	BL	3.5
8	LRI 008	NR	29	Male	MB	LL	4
9	LRI 009	T2R	32	Female	MB	LL	4.25
10	LRI 010	NR	52	Male	MB	BL	3
11	LRI 011	T1R	40	Male	PB	TT	0
12	LRI 012	T1R	47	Male	MB	BT	0.25
13	LRI 013	NR	50	Male	MB	BT	0
14	LRI 014	T2R	35	Female	MB	LL	4
15	LRI 015	NR	17	Female	MB	LL	3
16	LRI 016	T2R	60	Male	MB	LL	4
17	LRI 017	NR	42	Male	MB	BT	0
18	LRI 018	NR	35	Female	MB	LL	4.5
19	LRI 019	NR	35	Male	MB	BL	3.25
20	LRI 020	T2R	52	Male	MB	LL	4
21	LRI 021	T1R	26	Male	MB	BT	0
22	LRI 022	NR	25	Male	MB	BT	0
23	LRI 023	NR	29	Male	MB	BT	0
24	LRI 024	NR	33	Male	MB	BT	0
25	LRI 025	T1R	30	Female	MB	BT	0
26	LRI 026	NR	27	Female	PB	BT	0
27	LRI 027	T1R	35	Male	MB	BL	2.5
28	LRI 028	T2R	35	Male	MB	LL	4.75
29	LRI 029	NR	58	Male	MB	LL	3.25
30	LRI 030	NR	18	Female	MB	BL	1.5
31	LRI 031	NR	42	Male	MB	LL	3
32	LRI 032	T2R	35	Female	MB	BL	4.25
33	LRI 033	T1R	20	Male	MB	BL	2.25
34	LRI 034	NR	28	Male	MB	BT	0
35	LRI 035	NR	29	Female	MB	BL	1
36	LRI 036	T1R	30	Female	MB	BL	4
37	LRI 037	T1R	43	Male	MB	BT	0.25
38	LRI 038	T1R	27	Male	MB	LL	4
39	LRI 039	T1R	61	Male	MB	BB	1
40	LRI 040	T1R	42	Male	MB	BT	0
41	LRI 041	T1R	32	Male	PB	BT	0
42	LRI 042	T2R	35	Male	MB	LL	4
43	LRI 043	T1R	36	Female	MB	BT	0
44	LRI 044	T1R	55	Male	MB	BT	0
45	LRI 045	T1R	40	Female	MB	LL	2.75
46	LRI 046	T2R	82	Male	MB	LL	3
47	LRI 047	T1R	52	Male	MB	BL	2.25
48	LRI 048	T1R	24	Female	MB	BT	0
49	LRI 049	T2R	4	Female	MB	LL	3
50	LRI 050	T2R	21	Female	MB	LL	3
51	LRI 051	T2R	32	Male	MB	LL	3.5
52	LRI 052	T2R	35	Male	MB	LL	4
53	LRI 053	T2R	24	Female	MB	LL	3
54	LRI 054	T2R	22	Female	MB	LL	3
55	LRI 055	T2R	28	Male	MB	BL	2.35
56	LRI 056	T2R	30	Female	MB	LL	3+
57	LRI 057	T2R	37	Male	MB	LL	3.75
58	LRI 058	T2R	19	Male	MB	LL	3
59	LRI 059	T2R	32	Male	MB	LL	3.5
60	LRI 060	T2R	26	Male	MB	LL	6

### *Mycobacterium Leprae* whole transcriptome hybridization arrays

Total RNA was extracted following the Trizol protocol (Qiagen RNeasy Lipi Tissue kit—Cat#74804) and bacterial RNA was enriched in the samples. The quality of RNA was estimated using BioAnalyzer 2,100 (Agilent Technologies) followed by labelling, reverse transcription, amplification and hybridization to the arrays. Based on the BioAnalyzer reports for RNA quality estimations,16 NR, 18 T1R and 19 T2R samples were found suitable for whole transcriptome hybridizations.

A 2x400K gene expression array (whole-genome tiling array) was designed with the probes having 60-mer oligonucleotides tiling every 10 bp of the genome sequence of *M. leprae* (NC_011896.1) by Genotypic Technology Pvt. Ltd. (Bengaluru, India). The array comprised 420,288 features which include probes and Agilent controls. The samples for gene expression were labelled using the Agilent Quick-Amp labelling Kit (p/n5190-0442). 500 ng each of total RNA was reverse transcribed at 40°C using oligo dT primer tagged to a T7 polymerase promoter and converted to double-stranded cDNA. Synthesized double-stranded cDNA were used as templates for cRNA generation. cRNA was generated by *in vitro* transcription and the dye Cy3 CTP (Agilent) was incorporated during this step. The cDNA synthesis and *in vitro* transcription steps were carried out at 40°C. Labelled cRNA was cleaned up using Qiagen RNeasy columns (Qiagen, Cat No: 74106) and quality was assessed for yields and specific activity using the Nanodrop ND-1000. The hybridized slides were scanned on a G2600D scanner (Agilent Technologies). The data thus acquired is analyzed using GeneSpring GX Version 12.1 software. Data were normalized and a fold difference in expression was noted from 359,922 probes which include sense and antisense orientations of 179,961 probes. The differentially expressing *M. leprae* genomic regions between type 1, type 2 reactions and non-reactional cases were noted. All the samples were performed in technical replicates to validate the observations and microarray data corresponding to 359,922 probes for each of the samples (Figure 1). The data as well as the array design was uploaded to the Gene Expression Omnibus (GEO) Repository of the National Center for Biotechnology Information (NCBI) with the accession numbers: GSE85948 and GPL22363.

The 75th percentile ranking was used to normalize the probe intensities. The fold difference in expression was noted by subtracting the gene intensities of reactional samples from that of non-reactional samples in each experiment using the geometric mean of the technical replicates. These fold changes were log-transformed to base 2 and volcano plots were generated to identify differentially expressed genes (DEGs). The fold change of ≥ 0.6 was considered as upregulated and ≤ − 0.6 was considered as down-regulated. The Benjamin Hochberg adjusted *p* values were represented as –log_10_ (*p* value; Rajkumar et al., 2015).

### RT^2^ PCR profiler arrays

RT^2^ PCR profiler arrays (Qiagen Inc., United States) for human inflammatory cytokines and receptors (PAHS-011Z) were used to quantitate expression levels of 96 human immune genes in the lesional skin RNA samples across the study group. Each catalogued RT^2^Profiler PCR Array contains a list of the human inflammatory cytokines and receptors genes as well as five housekeeping (reference) genes on the array. In addition, each array contains a panel of proprietary controls to monitor genomic DNA contamination (GDC) as well as the first strand synthesis (RTC) and real-time PCR efficiency (PPC). The list of genes was provided in Qiagen array Cat. no. PAHS-011Z. Total RNA was isolated from skin biopsy specimens using RNeasy kit (Qiagen Cat No: Cat. No./ID: 74104) according to the manufacturer’s instructions. RNA quality was determined using a Nanodrop and was reverse transcribed using a QuantiTect Reverse Transcription Kit (Cat No: Cat. No./ID: 205311). The cDNA was used on the real-time RT^2^ Profiler PCR Array (Cat. no. PAHS-011Z) in combination with RT^2^SYBR Green qPCR Mastermix (Qiagen Cat. no. 330529). Fold-change values greater than one indicates a positive- or an up-regulation, and the fold-regulation is equal to the fold-change. Fold-change values less than one indicate a negative or down-regulation, and the fold-regulation is the negative inverse of the fold-change. The *value of ps* were calculated based on a Student’s *t*-test of the replicate 2^(−Delta Ct)^ values for each gene in the control group and treatment groups. The data was analyzed using Qiagen GeneGlobe application for RT^2^ PCR profiler arrays.

### Multiplex qPCR

RNA extracted from lesional skin biopsy specimens (NR = 16; T1R = 16; T2R = 9) was used in the qPCR assays. The numbers were different from the size of the study groups as these are the samples in which RNA quality is optimal for cDNA preparation and qPCR. The genes GNLY, CD8A, CXCL10, IFI6, IL10, PRF1, CCL2, FCGR1B, OAS1, IFI44, and CTLA4 have been amplified using the conditions described elsewhere (Supplementary Material-1; Supplementary Table S3; Tió-Coma et al., 2021). The data of the qPCR were analyzed using Thermo Fisher Cloud and GraphPad Prism version 8.0.1. Samples were analyzed in duplicates and ΔCts were calculated using GAPDH as the reference gene. Mann–Whitney *U*-test was performed to determine if differences in gene expression is statistically significant.

### Statistical analysis

For the transcriptome data, normalization of DEGs and the expression threshold of ± 0.6 for log_2_fold_change (log_2_FC) was determined using Agilent Gene Spring GX software (Agilent Inc.; Supplementary Table 1). The principal component analysis was performed using in-built prcomp () function in R and plots were generated using ggplot2 package. The functional GO term enrichment analysis was performed using DAVID database (Sherman et al., 2022) and software.

## Results

### Analysis of transcriptome-wide changes using principal component analysis

**Figure 2:**
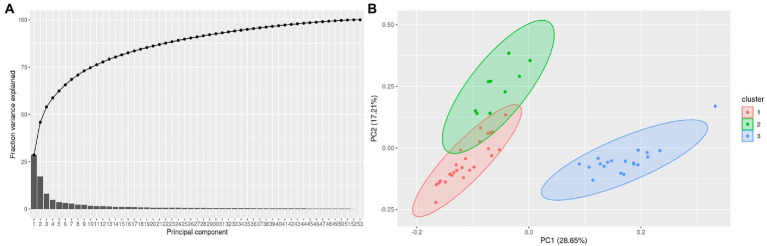
**(A)** A pareto plot with 80% of the fraction variance explained by the first 5 PCs using PCs for each of the sample and **(B).** Clusters mapped between PC1 and PC2 in a 2-dimentional PCA plot. The clusters contained 16 non reactional controls (NR), 18 T1R and 19 T2R samples.

Considering 1,600 genes and 45 rRNA transcripts whose intensities were noted from the hybridization arrays, the normalized data with log_2_FC values were subjected to principal component (PC) analysis to estimate the sample variance and reduce the dimensionality in the data. We first determined if the number of PCs are sufficient to explain the fraction of variance using a Pareto chart. A sequential reduction in variance across PCs was noted with PC 53 being zero indicating that the sample numbers are sufficient to explain variance (Figure 2A). Further we visualized the clusters using ggfortify () package in R and plotted the clusters from the PCs (Figure 2B).

Cluster 1 largely represents NR, Cluster 2 T1R, Cluster 3 T2R samples (Figure 2B). Given the diverse Ridley Jopling (RJ) and bacteriological index (BI) classification across the NR samples, we did a differential gene expression analysis of the NR samples that have a BI of zero in comparison to those that have positive BI. We noted that genes ML0247(putative arsenate reductase), ML2269 (putative hydrolase), ML2296 (putative membrane protein) and ML1182(PPE-family protein) are over expressed in BI-zero NR samples whereas ML1466 (50S ribosomal protein L27) and ML1180 (Putative ESAT-6-like protein X) are upregulated in BI positive samples (Supplementary Material-1; Supplementary Figure S1).

### Differentially expressed genes of *Mycobacterium leprae* across the reactional states

**Figure 3:**
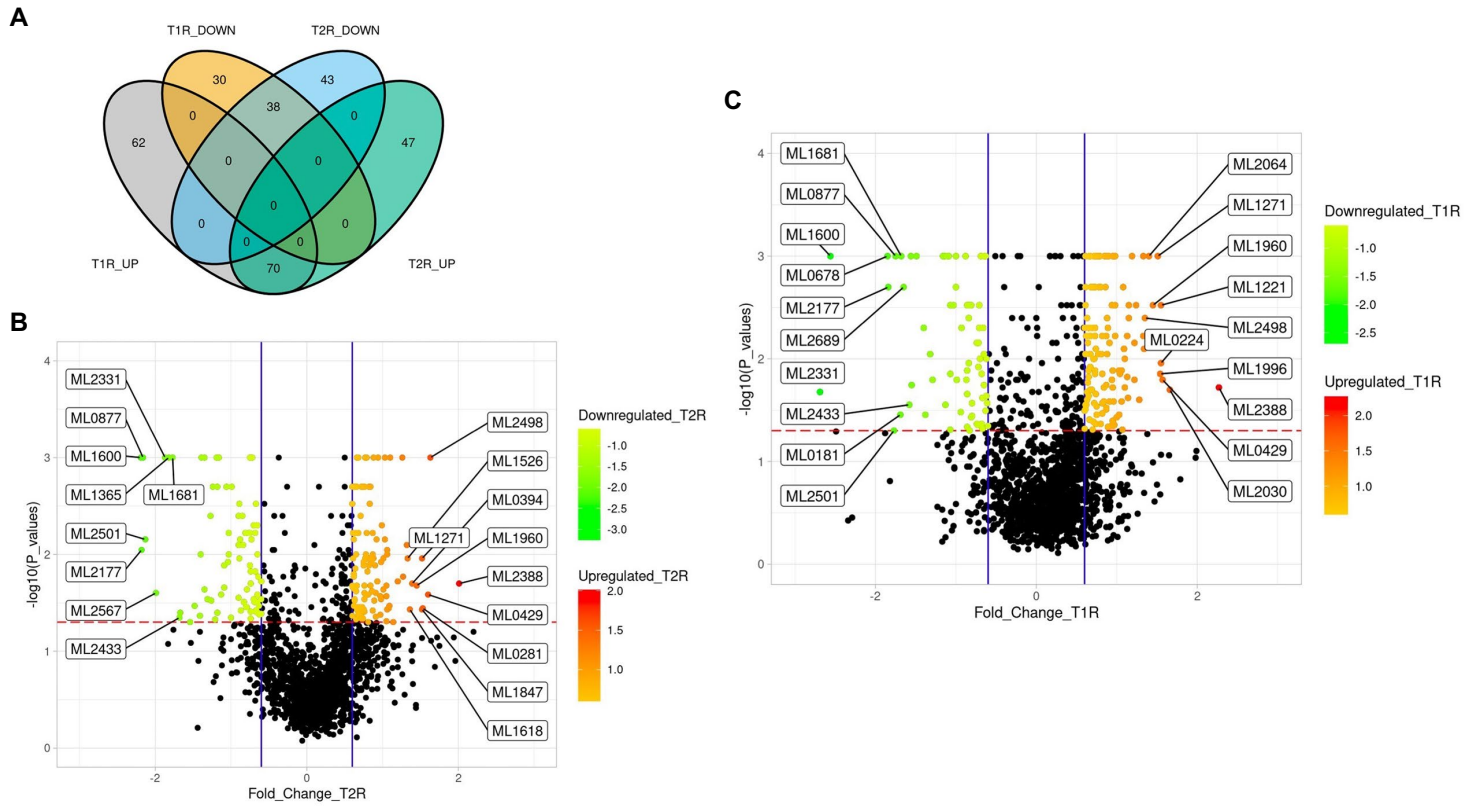
**(A)** Venn diagram representing the number of significantly upregulated and downregulated genes of *Mycobacterium leprae* across the study groups. **(B,C)** Volcano plots with log_2_FC (derived from normalized gene expression data) showing the top 10 differentially expressed genes in T1R and T2R compared to NR cases. The overexpressed genes are those with the volcano plot *p*-values < 0.05 and Log_2_FC values of ≥ 0.6 (mean of each of the reaction groups) and underexpressed genes are those with the volcano plot *p*-values < 0.05 and Log_2_FC values of ≤ − 0.6 (for the mean of each of the reaction groups). Scales in **B** and **C** represent the Log_2_FC values.

From the log_2_FC values, we noted transcripts corresponding to 132 genes of *M. leprae* for T1R and 117 genes in T2R that are significantly upregulated in comparison to NR. In both the reactional states, 70 genes were upregulated, and 38 genes were downregulated (Figure 3A). Benjamin Hochberg adjusted *value of ps* were < *0.05* for all these associations. We identified only one gene (ML2664) that was upregulated in T1R and downregulated in T2R and no DEGs in converse (Supplementary Table 2). The top 10 upregulated and the lower 10 downregulated genes were labelled in the volcano plots in Figures 3B,C. The NR sample was further split based on the Ridley Joplin classification into two groups—the TT/BT group and the BL/LL group. The TT/BT group was compared with T1R and BL/LL with T2R. We noted that the top 10 DEGs are retained the same as for the whole data set even after splitting the control sample based on RJ classification (Supplementary Material-1; Supplementary Figure S2).

### Unsupervised clustering analysis for expression patterns

**Figure 4:**
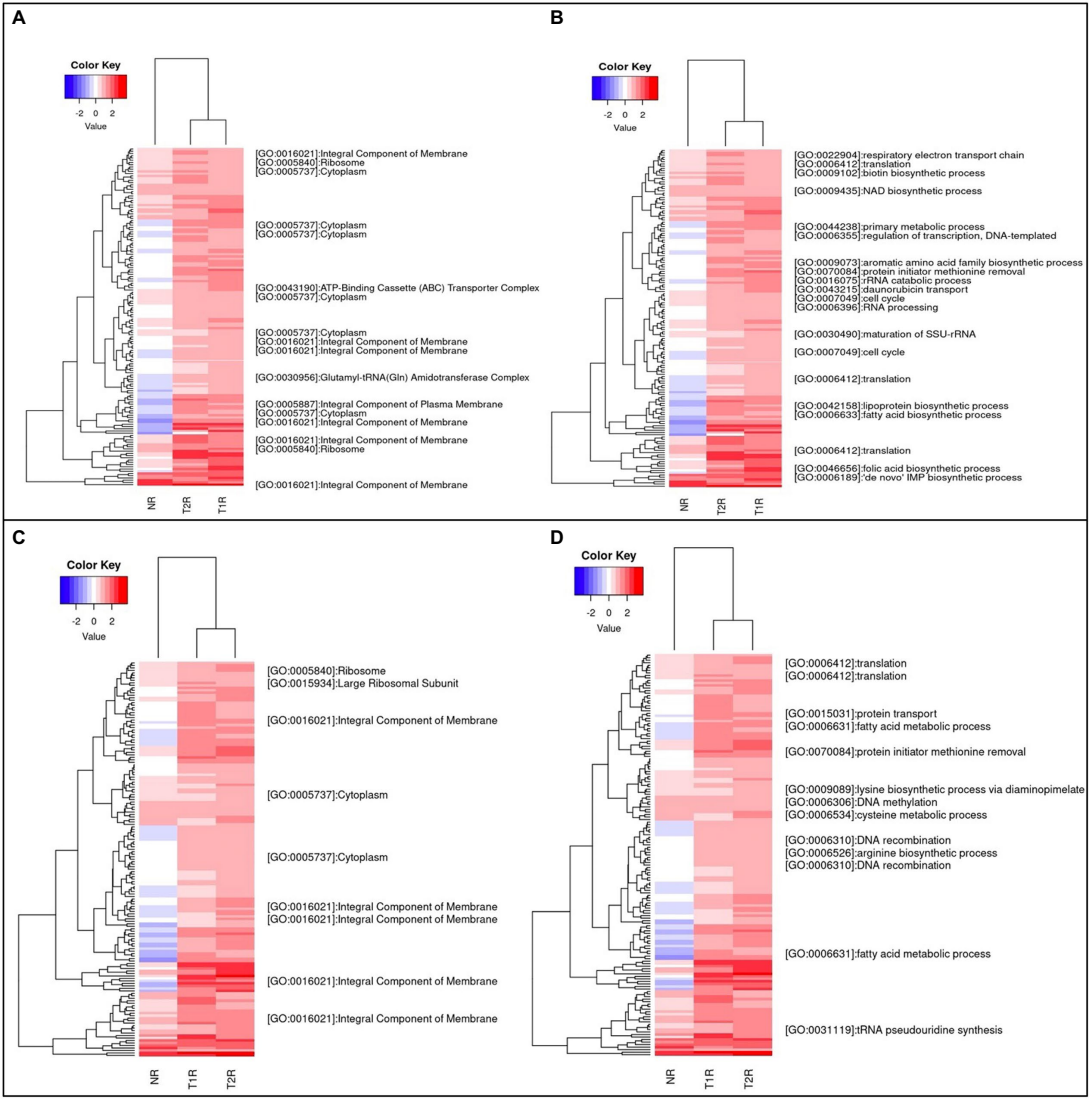
**(A,B)** represent heatmap of the genes that are upregulated in T1R in comparison to NR and **(C,D)** includes genes that are upregulated in T2R in comparison to NR. All the genes listed are significantly overexpressed (*p* < 0.05) and the color key represents the Log2FC values for each gene ranging from −2 to 2. The blank lines in GO terms are genes without GO annotations. In both the figures, we retained both T1R and T2R gene expressions to visualize expression changes across the sample types.

Hierarchical clustering analysis with the *z* scores of the fold changes gene-wise across the study groups revealed clusters with various enriched GO terms. Among the upregulated genes in T1R, we noted over representation of genes that encode integral membrane proteins, followed by cytosolic and ribosome bound protein coding genes (Figure 4A). From the GO biological processes genes corresponding to proteins that mediate cell wall biosynthesis, lipid biosynthetic pathways, drug transport, fatty acid and amino acid metabolism, and the biotin and folic acid biosynthetic pathways are overrepresented (Figure 4B). In the T2R, among the genes that had GO annotations for cellular component, those that encode integral membrane components were higher in number (Figure 4C) and from the GO biological processes (Figure 4D), genes involved in translation, DNA recombination, fatty acid metabolism, protein transport and amino acid metabolism are present.

### Functional term analysis from GO annotations

**Figure 5:**
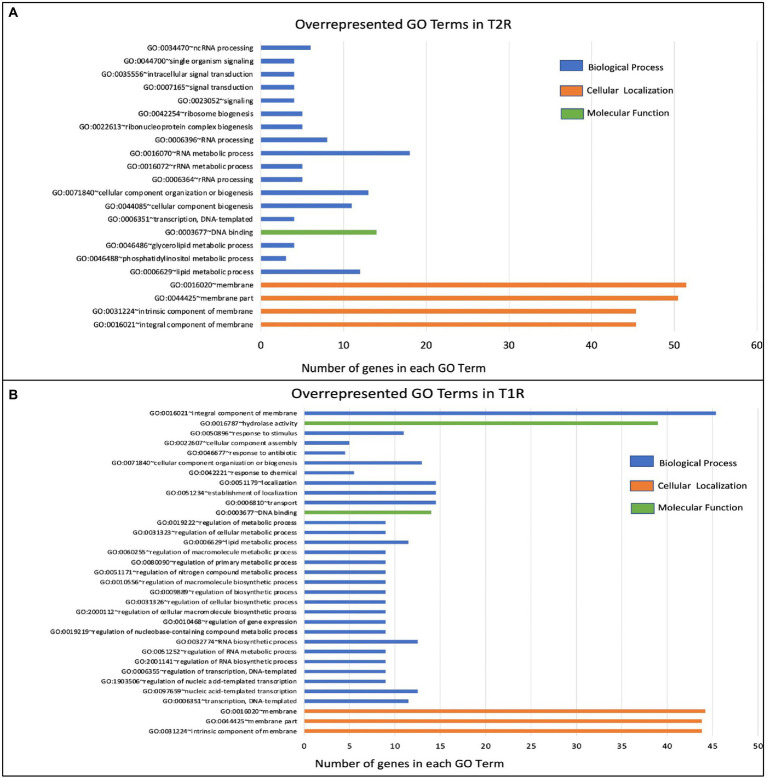
Over-represented GO terms in the T1R Group (*p* < 0.05) in comparison to NR **(A)** and in the T2R Group (*p* < 0.05) in comparison to NR group **(B)**. The number of genes on *x*-axis indicates genes that have the same GO term in each of the classes (biological process, cellular localization, and molecular function).

We further used the enriched GO terms from the DAVID database to ascertain the probability of gene co-occurrence in each GO term across the sample. Among the upregulated genes in T1R in comparison to NR, for GO cellular component terms for integral component of membrane [GO:0016021], intrinsic component of membrane [GO:0031224], the membrane part [GO:0044425] and membrane [GO:0016020] were significantly overrepresented (*p-value 0.0004*). In the GO biological processes, terms for transcription and regulation of transcription, RNA biogenesis, regulation of RNA biosynthetic processes and lipid metabolism were overrepresented among others noted in Table 2 and Figure 5A. For the GO Molecular Function, genes involved in DNA binding and hydrolase activity are noted. For genes that are overexpressed in T2R in comparison to NR, the GO cellular component terms for integral component of membrane [GO:0016021], intrinsic component of membrane [GO:0031224], the membrane part [GO:0044425] and membrane [GO:0016020] were significantly overrepresented (*p-value 0.008*). For the Biological Process terms, lipid metabolic processes, cellular component biogenesis, RNA processing, RNA metabolic process, ribonucleoprotein complex biogenesis and genes involved in signal transduction were significantly overexpressed along with others shown in Table 2 and Figure 5B.

**Table 2:** The enriched GO terms in T1R and T2R groups across the sample.

GO term	GO type	Reaction type	Count^*^	*p*-value
GO:0016021 ~ integral component of membrane	Cellular Localization	T1R	44	0.000041
GO:0031224 ~ intrinsic component of membrane	Cellular Localization	T1R	44	0.000045
GO:0044425 ~ membrane part	Cellular Localization	T1R	44	0.000060
GO:0016020 ~ membrane	Cellular Localization	T1R	44	0.000197
GO:0006351 ~ transcription, DNA-templated	Biological Process	T1R	12	0.021228
GO:0097659 ~ nucleic acid-templated transcription	Biological Process	T1R	13	0.011823
GO:1903506 ~ regulation of nucleic acid-templated transcription	Biological Process	T1R	9	0.005321
GO:0006355 ~ regulation of transcription, DNA-templated	Biological Process	T1R	9	0.005321
GO:2001141 ~ regulation of RNA biosynthetic process	Biological Process	T1R	9	0.005321
GO:0051252 ~ regulation of RNA metabolic process	Biological Process	T1R	9	0.006193
GO:0032774 ~ RNA biosynthetic process	Biological Process	T1R	13	0.017436
GO:0019219 ~ regulation of nucleobase-containing compound metabolic process	Biological Process	T1R	9	0.008264
GO:0010468 ~ regulation of gene expression	Biological Process	T1R	9	0.009478
GO:2000112 ~ regulation of cellular macromolecule biosynthetic process	Biological Process	T1R	9	0.010821
GO:0031326 ~ regulation of cellular biosynthetic process	Biological Process	T1R	9	0.010821
GO:0009889 ~ regulation of biosynthetic process	Biological Process	T1R	9	0.010821
GO:0010556 ~ regulation of macromolecule biosynthetic process	Biological Process	T1R	9	0.010821
GO:0051171 ~ regulation of nitrogen compound metabolic process	Biological Process	T1R	9	0.013930
GO:0080090 ~ regulation of primary metabolic process	Biological Process	T1R	9	0.015711
GO:0060255 ~ regulation of macromolecule metabolic process	Biological Process	T1R	9	0.015711
GO:0006629 ~ lipid metabolic process	Biological Process	T1R	12	0.029266
GO:0031323 ~ regulation of cellular metabolic process	Biological Process	T1R	9	0.017654
GO:0019222 ~ regulation of metabolic process	Biological Process	T1R	9	0.017654
GO:0003677 ~ DNA binding	Molecular Function	T1R	14	0.048031
GO:0006810 ~ transport	Biological Process	T1R	15	0.031141
GO:0051234 ~ establishment of localization	Biological Process	T1R	15	0.031141
GO:0051179 ~ localization	Biological Process	T1R	15	0.031141
GO:0042221 ~ response to chemical	Biological Process	T1R	6	0.012239
GO:0071840 ~ cellular component organization or biogenesis	Biological Process	T1R	13	0.037976
GO:0046677 ~ response to antibiotic	Biological Process	T1R	5	0.021659
GO:0022607 ~ cellular component assembly	Biological Process	T1R	5	0.044536
GO:0050896 ~ response to stimulus	Biological Process	T1R	11	0.035335
GO:0016787 ~ hydrolase activity	Molecular Function	T1R	39	0.045506
GO:0016021 ~ integral component of membrane	Cellular Localization	T2R	45	0.008044
GO:0031224 ~ intrinsic component of membrane	Cellular Localization	T2R	45	0.008261
GO:0044425 ~ membrane part	Cellular Localization	T2R	50	0.003330
GO:0016020 ~ membrane	Cellular Localization	T2R	51	0.007984
GO:0006629 ~ lipid metabolic process	Biological Process	T2R	12	0.036602
GO:0046488 ~ phosphatidylinositol metabolic process	Biological Process	T2R	3	0.037410
GO:0046486 ~ glycerolipid metabolic process	Biological Process	T2R	4	0.044399
GO:0003677 ~ DNA binding	Molecular Function	T2R	14	0.027195
GO:0006351 ~ transcription, DNA-templated	Biological Process	T2R	4	0.044833
GO:0044085 ~ cellular component biogenesis	Biological Process	T2R	11	0.002047
GO:0071840 ~ cellular component organization or biogenesis	Biological Process	T2R	13	0.002468
GO:0006364 ~ rRNA processing	Biological Process	T2R	5	0.006889
GO:0016072 ~ rRNA metabolic process	Biological Process	T2R	5	0.006889
GO:0016070 ~ RNA metabolic process	Biological Process	T2R	18	0.007569
GO:0006396 ~ RNA processing	Biological Process	T2R	8	0.008240
GO:0022613 ~ ribonucleoprotein complex biogenesis	Biological Process	T2R	5	0.032265
GO:0042254 ~ ribosome biogenesis	Biological Process	T2R	5	0.032265
GO:0023052 ~ signaling	Biological Process	T2R	4	0.034417
GO:0007165 ~ signal transduction	Biological Process	T2R	4	0.034417
GO:0035556 ~ intracellular signal transduction	Biological Process	T2R	4	0.034417
GO:0044700 ~ single organism signalling	Biological Process	T2R	4	0.034417
GO:0034470 ~ ncRNA processing	Biological Process	T2R	6	0.046209

* Count indicates the number of genes in each GO term class. These are the genes that has a log_2_FC of > 0.6.

### Expression profiles of known *Mycobacterium leprae* antigens from the IEDB database

**Figure 6:**
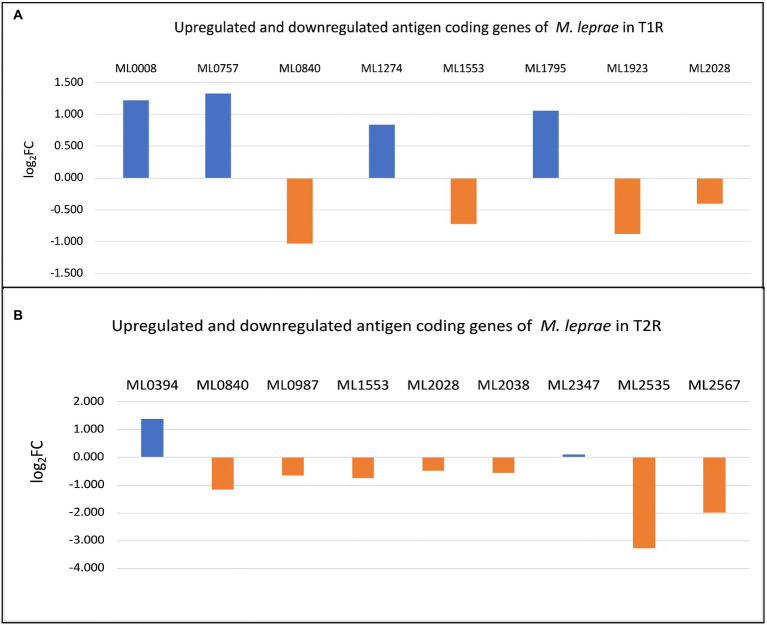
**(A)** Differentially expressed genes (DEGs) among antigen coding genes in T1R. **(B)** DEGs among antigen coding genes in T2R.

From the 61 antigens recorded in Immune Epitope Database (IEDB) for *M. leprae* (identified by the search term *Bacillus leprae*), we selected 58 protein antigens which has Uniprot IDs (Table 3) and studied their expression across the sample. The significantly upregulated antigen coding genes in T1R and T2R are depicted in Figures 6A,B, respectively.

**Table 3:** The log_2_FC for DEGs among the antigen coding genes in immune epitope database (IEDB) database for *Mycobacterium leprae.*

Gene name	FC_NR	FC_T1R	FC_T2R	*p*-value_T1R	*p*-value_T2R
ML0008	0.060	1.220	0.830	0.0157	0.0667
ML0050	0.210	0.420	1.100	0.6053	0.2289
ML0091	−0.020	−0.270	−0.140	0.2622	0.2371
ML0098	0.020	−0.430	−0.160	0.1750	0.5904
ML0126	0.100	0.310	0.390	0.0792	0.1099
ML0136	0.030	0.470	0.530	0.3796	0.2864
ML0176	0.020	−0.230	−0.400	0.2519	0.2946
ML0234	−0.340	−0.280	−0.160	0.5852	0.6195
ML0308	0.020	0.010	−0.040	0.2947	0.1061
ML0317	0.070	0.130	0.010	0.5024	0.0667
ML0375	−0.030	0.300	0.280	0.4125	0.1388
ML0380	0.080	−0.050	0.630	0.4594	0.2821
ML0394	0.170	1.190	1.390	0.1120	0.0198
ML0398	0.010	−0.110	−0.020	0.2699	0.3885
ML0411	0.010	−0.160	−0.010	0.5904	0.7327
ML0576	0.080	0.150	0.020	0.1918	0.2723
ML0611	0.000	−0.080	−0.230	0.1740	0.2657
ML0638	0.060	−0.350	−0.460	0.1855	0.0651
ML0726	0.000	−0.030	−0.280	0.0816	0.1436
ML0757	0.060	1.330	0.940	0.0006	0.1158
ML0840	−0.030	−1.030	−1.160	0.0028	0.0004
ML0841	0.020	0.570	0.420	0.0792	0.3072
ML0885	0.040	0.360	0.560	0.0572	0.0869
ML0987	−0.070	−0.590	−0.650	0.1609	0.0398
ML1057	0.040	0.140	−0.020	0.1972	0.2291
ML1189	0.040	−0.280	−0.050	0.4162	0.4799
ML1207	−0.080	−0.460	−0.680	0.0641	0.0671
ML1214	0.030	0.370	0.250	0.2873	0.3489
ML1217	−0.020	0.260	0.250	0.2007	0.1549
ML1274	−0.080	0.840	0.960	0.0265	0.0683
ML1358	−0.030	0.540	0.470	0.1187	0.1509
ML1419	−0.060	−0.480	−0.670	0.4055	0.0604
ML1420	0.000	−0.150	−0.140	0.2311	0.2457
ML1553	−0.060	−0.720	−0.740	0.0067	0.0146
ML1601	−0.020	−0.150	0.000	0.2092	0.3392
ML1795	0.070	1.060	0.970	0.0258	0.0594
ML1811	0.100	0.000	−0.090	0.3448	0.3400
ML1812	0.010	−0.130	0.320	0.4159	0.4812
ML1829	0.000	0.310	0.390	0.3728	0.1712
ML1891	−0.070	0.070	0.160	0.3920	0.4598
ML1915	0.010	0.070	0.090	0.1735	0.1664
ML1923	−0.050	−0.880	−0.950	0.0004	0.0532
ML1989	0.000	0.410	0.510	0.0749	0.0581
ML1990	−1.060	0.690	1.400	0.2225	0.2180
ML2028	−0.050	−0.400	−0.480	0.0254	0.0399
ML2038	0.000	−0.430	−0.550	0.0787	0.0299
ML2055	0.060	0.320	0.890	0.2013	0.0559
ML2069	−0.010	0.090	0.010	0.3061	0.1957
ML2283	0.290	0.470	−0.210	0.4902	0.3048
ML2347	0.040	−0.300	0.100	0.2942	0.0083
ML2395	0.110	0.440	0.280	0.3971	0.5247
ML2400	0.040	0.240	0.330	0.1476	0.1593
ML2452	−0.020	0.520	0.490	0.0712	0.0858
ML2496	−0.120	0.040	−0.070	0.3343	0.1724
ML2531	0.260	0.610	0.490	0.6400	0.4597
ML2535	−0.340	−2.490	−3.260	0.0513	0.0092
ML2567	0.060	−1.880	−1.990	0.0525	0.0245
ML2688	−0.020	0.020	0.100	0.3925	0.4782

### Consistent overexpression of ML2388 across the sample in reactional states of leprosy

**Figure 7:**
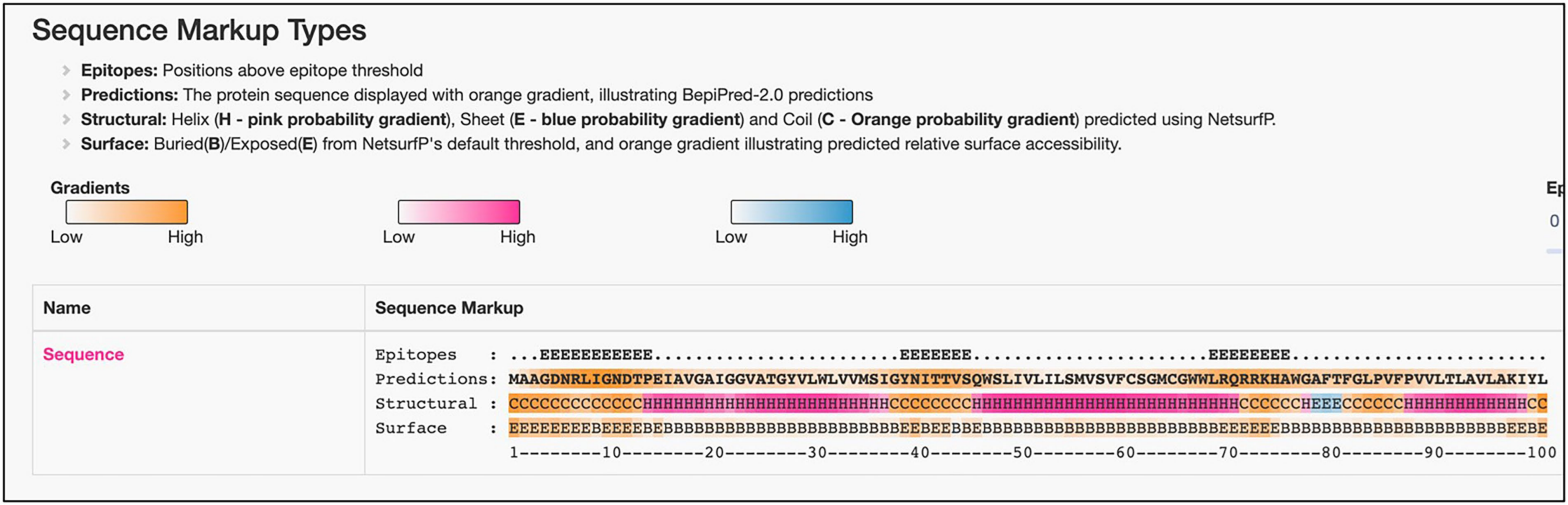
Predicted linear B-Cell epitopes in the possible membrane protein—ML2388.

Among all the significant DEGs noted across the sample, we have seen consistent over expression of ML2388 across the reactional states T1R and T2R in comparison to NR. This gene encodes a possible membrane protein and has GO term for cellular localization as the integral component of the membrane. We predicted the presence of linear B cell epitopes in this protein using Bepipred 2.0. The results were presented in Figure 7. Three linear epitopes were predicted with high confidence in the exposed and helical or coiled regions of the protein (Reece et al., 2006).

### Differential expression of human immune genes

From the RT2 PCR Profiler arrays, the expression data was analyzed using Qiagen GeneGlobe pipeline using the ∆∆Ct method. The threshold cycle values across the study groups were normalized using the house keeping gene [GAPDH (Glyceraldehyde-3-phosphate dehydrogenase)] expression levels (Kwittken, 1968). The quality checks were performed by measuring the PCR array reproducibility, ∆Ct of the reverse transcription control and genomic DNA contamination. For the test sets (T1R and T2R), the PPC was 15 which indicates that the array reproducibility check has been passed, the transcription control has a ∆Ct of 5.28 and ∆Ct of genomic DNA contamination is 33 which indicates that the contamination is minimal. The cut off Ct value is set to 33. Fold-Change (2^(−∆∆Ct)^) is the normalized gene expression (2^(−∆Ct)^) in the Test Sample divided the normalized gene expression (2^(− ∆Ct)^) in the Control Sample. Fold-Regulation represents fold-change results in a biologically meaningful way.

We noted a significant upregulation of CXC chemokines, CXCL9, CXCL10, CXCL2, CXCL11, CD40 ligand (CD40LG), and interleukin IL17A in T1R. In T2R, CXC chemokines, CXCL10, CXCL11, CXCL9, CXCL2 and CD40 ligand (CD40LG) were upregulated (Scollard et al., 2011).

### Multiplex qPCR assays

**Figure 8:**
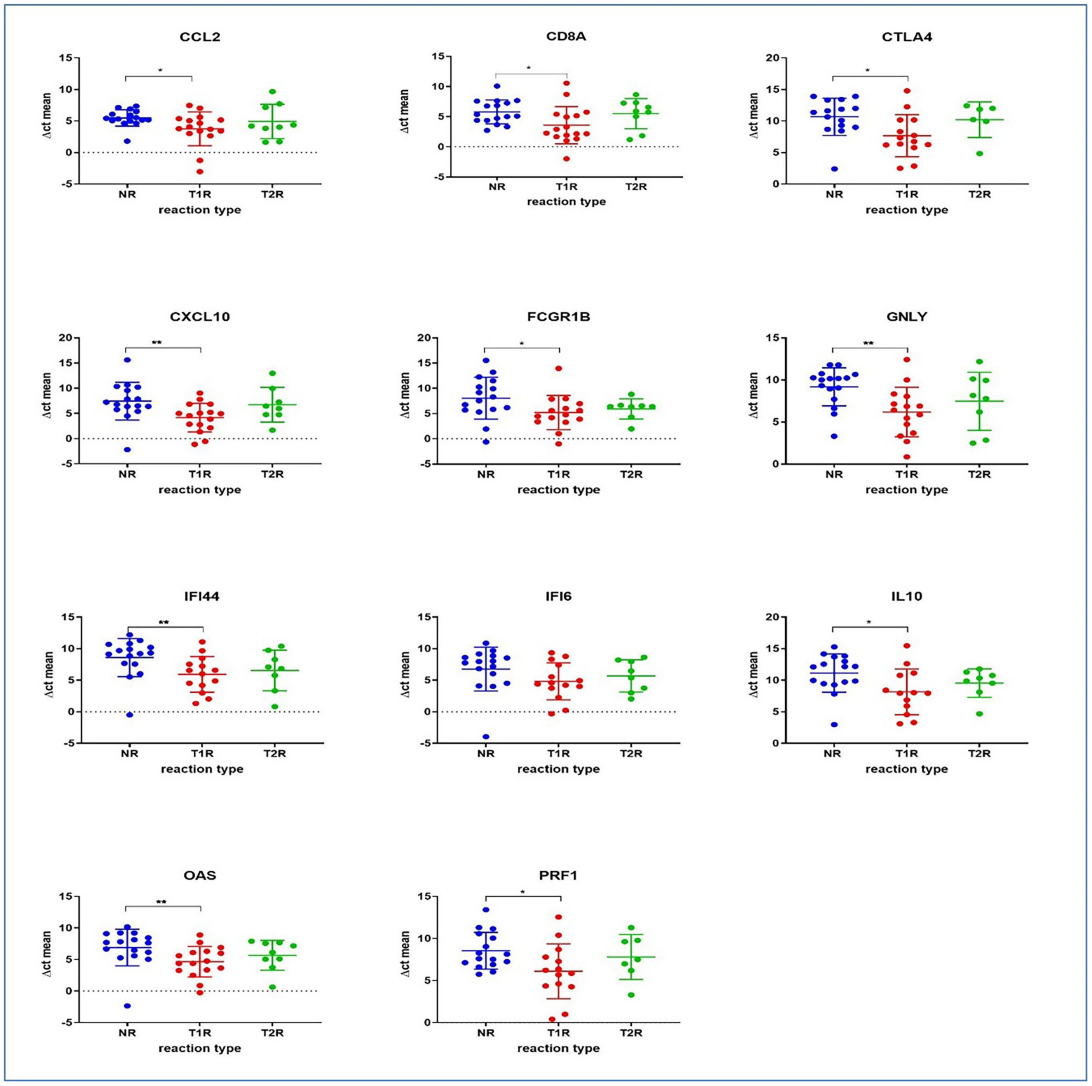
The panel depicts immune gene expressions across the study groups as determined by qPCR. Statistically significant differences in mean of the ∆Ct for technical replicates of each of the sample was denoted by ^*^ for value of *p* of 0.05 and ^**^ for value of *p* of 0.005.

We also tested the expression levels of a selected set of human immune genes that were observed elsewhere (Teles et al., 2013; Geluk et al., 2014; Tió-Coma et al., 2019) to have associations with reactional states in leprosy using the multiplex qPCR system in our study groups [T1R (*n* = 16), T2R (*n* = 9) and NR (*n* = 16)]. Between the NR and the T1R groups, a significant difference in expression was observed for genes GNLY (Granulysin), CD8A, CXCL10, IL10, PRF1 (Perforin 1), CCL2, FCGR1B (Fc Gamma Receptor Ib), OAS1 (2′-5’-Oligoadenylate Synthetase 1), IFI44 (Interferon Induced Protein 44) and CTLA4 (cytotoxic T-lymphocyte-associated protein 4). Gene expression was higher (lower ∆Ct) for all the genes tested in patients with T1R than NR patients. No statistically significant differences in expression were noted between NR and T2R or T1R and T2R (Figure 8).

## Discussion

Reactional states in leprosy pose a significant challenge to the global efforts to contain the disease. These immune exacerbations are a major cause of nerve function impairment and consequent disabilities in leprosy. Various host-related factors have been reported as risk factors for T1R and these include age, severity of the disease, and having a positive BI in the slit skin smears. Lepromatous leprosy with the BI of more that 4 + is identified as a risk factor for T2R in leprosy (Pandhi and Chhabra, 2013). Few studies have also implicated antigenic triggers in Type 1 reaction, leading to expansion of both cross-reactive and specific T-cells. Despite limited genotypic variability between various strains of *M. leprae*, the patterns of infection, pathogenicity and virulence largely differ across various clinical phenotypes. This suggests that success of infection and leprosy progression is largely dependent on host′s immune response and genetic complement (Texereau et al., 2005). Studies on *M. leprae* and host immune gene expression signatures can help identify potential biomarkers to predict reactional states in leprosy. In this study, we conducted a case–control association analysis of DEGs in *M. leprae* transcriptome in the lesional skin tissues of leprosy cases in T1R and T2R and compared them with those without any hypersensitivity reactions to decipher a gene expression signature for these reactional states. From the GEO datasets for *M. leprae*, while there are several studies on understanding host transcriptomic response (Teles et al., 2013; Manry et al., 2017; Zhang et al., 2019; Leal-Calvo et al., 2021) to *M. leprae* infection, we identified only one other study (Montoya et al., 2019) that profiled differential gene expression within *M. leprae* transcriptome to ascertain their implications on reactional states. Although, our experiments were conducted with total RNA, we limited our analysis to expression profiles in the protein coding genes. While identifying DEGs across study groups remained the main objective, we attempted to delineate the possible functional implications of DEGs on the reactional outcomes. The gene chip array from Agilent that we employed in the experiments is available at the GEO repository (GPL22363).

From the upregulated genes in T1R (*n* = 132), genes ML2064, ML1271, ML1960, ML1220, ML2498, ML1996, ML2388, ML0429, ML2030 and ML0224 are the top 10 genes with highest Log2FC values. Most of these genes code for integral or intrinsic membrane proteins while others encode proteins of the 30S ribosomal subunit and enzymes involved in glycolysis and possible enoyl-CoA hydratases. Additionally, we noted 27 genes that encode integral and intrinsic components of the cell wall and plasma membrane (Supplementary Table S2). We noted 12 virulence genes among the upregulated which include ML0114—an ABC O-antigen transporter, ML1014—RNA polymerase sigma factor sigB, ML1076-RNA (Williams et al., 2004 2007) polymerase sigma factor SigE, ML1128—Diaminopimelate decarboxylase, ML1220- Biotin synthase (Lastória and de Abreu, 2014), ML1547–4′-phosphopantetheinyl transferase, ML1656–3-oxoacyl-[acyl-carrier-protein] synthase, ML1675–Uracil-DNA glycosylase, ML2124–Sensor-type histidine kinase, ML2307–Transcriptional regulator and ML2350–ATP-dependent efflux pump essential for phthiocerol dimycocerosates translocation (Sharma et al., 2009; Orlova et al., 2013). Overexpression of virulence genes were noted in several other studies in reactional states (Kahawita and Lockwood, 2008; Nery et al., 2013). We also noted over expression of heat shock proteins (*hsp18*) in T1R (Lini et al., 2009).

In T2R, genes ML2498, ML1526, ML0394, ML1960, ML2388, ML0429, ML0281, ML1847, ML1618 and ML1271 were the Top-10 significantly upregulated genes. These encode conserved membrane proteins, proteins of the 30S and 50S ribosomal subunits, possible enoyl-CoA hydratases and other enzymes. In addition, we noted that 10 virulence genes were significantly over expressed. They are ML0243—Putative acyl-CoA synthetase, ML1014—RNA polymerase sigma factor sigB, ML1076—RNA polymerase sigma factor SigE, ML1128—Diaminopimelate decarboxylase, ML1633—Possible secreted hydrolase, ML1727—O-phosphoserine phosphohydrolase(Acebrón-García-de-Eulate et al., 2021), ML1925—Superoxide dismutase [Cu-Zn], ML1954—Pantothenate kinase(Hasan et al., 2004), ML2307—Transcriptional regulator and ML2439—Sensory transduction protein RegX3 (Duthie et al., 2007; Sapkota et al., 2010; Tang et al., 2021).

Among these we noted consistent over expression of ML2388. This gene encodes a putative membrane protein and possess three regions in the sequence that are predicted to be potential B-cell epitopes. To further understand the potential implications of previously characterized antigen coding genes in *M. leprae* with transcriptomic responses to reactions in leprosy, we measured their expression across the study groups and noted over-expression of integrase (ML0008), probable secreted protein (ML0757), phospotidylgycerol (ML1274) and 18Kda Heat shock Protein (ML1795) with T1R. In T2R, we noted overexpression of Methyltransf_25 domain-containing protein (ML0394).

From the host genes, we noted that between the NR and the T1R groups, a significant difference in expression was observed for genes GNLY (Granulysin) responsible for transfer of granzymes (a family of serine proteases traditionally known for their role in promoting cytotoxicity of foreign, infected or neoplastic cells), CD8A (Cytotoxic T-lymphocytes) important for immune defense against intracellular pathogens, including viruses and bacteria, CXCL10 (Interferon gamma induced protein-10). Granulysin has been noted to express high in tuberculoid lesions (Geluk et al., 2014). Upregulation of CD8 antigen in reversal reactions has also been noted in leprosy/HIV co-infections (de Oliveira et al., 2013).

Several studies revealed the upregulation of CXCL10 (IP10) in reactional states of leprosy (Chaitanya et al., 2013; van Hooij et al., 2016; Ferreira et al., 2021). Expression of IP-10 is seen in many Th1-type inflammatory diseases, where it is thought to play an important role in recruiting activated T cells into sites of tissue inflammation. Interleukin 10 (IL-10), a cytokine with potent anti-inflammatory properties, plays a central role in limiting host immune response to *M. leprae*, thereby preventing autoimmune inflammation and maintains tissue homeostasis. PRF1 (Perforin 1) is a glycoprotein responsible for pore formation in cell membranes of target cells and CCL2 chemokine which controls immunity by promoting regulatory T cell communication with dendritic cells in lymph nodes, are all upregulated in T1R. Further, FCGR1B (Fc Gamma Receptor Ib) which functions by binding to the Fc regions of immunglobulin, OAS1 (2′-5′-Oligoadenylate Synthetase 1) that helps in degradation of viral infections, IFI44 (Interferon Induced Protein 44) and CTLA4 (cytotoxic T-lymphocyte-associated protein 4) which acts as a halting mechanism and reduces the function of T cells are over expressed in T1R. Similar results were noted with RT2 profiler arrays in this study. CXC chemokines, CXCL9, CXCL10, CXCL2, CXCL11, CD40 ligand (CD40LG), and interleukin IL17A were upregulated in T1R. In T2R, CXC chemokines CXCL10, CXCL11, CXCL9, CXCL2 and CD40 ligand (CD40LG) were upregulated (Geluk et al., 2014; Teles et al., 2019; Tió-Coma et al., 2021).

In conclusion, we recommend bacterial genes ML2388, ML2664, and host immune genes CXCL10 and IL-17A as transcriptomic signatures for reactional states in leprosy. Our study profiled various upregulating and downregulating gene signatures from *Mycobacterium leprae* and human immune genes that demonstrated plausible association with reactional states in leprosy. Further studies are however required to validate the above identified gene expression signatures as predictive markers for leprosy reactions in a longitudinal cohort.

## Data availability statement

The datasets presented in this study can be found in online repositories. The names of the repository/repositories and accession number(s) can be found in the article/Supplementary material.

## Ethics statement

The studies involving human participants were reviewed and approved by Karigiri Research Committee. The patients/participants provided their written informed consent to participate in this study.

## Author contributions

MD, SV, and AG conceived the study. MT-C, DD, AV, and IH contributed to the data curation. MD, SV, and AV contributed to the formal analysis. SV and AG acquired funding. MD, SV, AV, DD, and MT-C investigated the study. SV, MD, and AG supervised the study. MD and SV wrote the original draft. AG, AV, and MT-C reviewed, and edited the manuscript. All authors reviewed, discussed, and agreed with the final manuscript.

## Funding

This work received the financial support from the Leprosy Research Initiative (LRI) and the Turing Foundation under LRI Grant number 704.16.57.

## Conflict of interest

The authors declare that the research was conducted in the absence of any commercial or financial relationships that could be construed as a potential conflict of interest.

## Publisher’s note

All claims expressed in this article are solely those of the authors and do not necessarily represent those of their affiliated organizations, or those of the publisher, the editors and the reviewers. Any product that may be evaluated in this article, or claim that may be made by its manufacturer, is not guaranteed or endorsed by the publisher.
