# Supplementary material for: Mycobacterium leprae and host immune transcriptomic signatures for reactional states in leprosy
Source: Front Microbiol. 2023 Mar 27;14:1113318. doi: 10.3389/fmicb.2023.1113318 (PMC10083373; doi:10.3389/fmicb.2023.1113318)
Supplement: Supplementary file 1 [file Data_Sheet_1.docx]

**Supplementary Material - 1**

Title: *Mycobacterium leprae* and host immune transcriptomic signatures for reactional states in leprosy.

**Authors:** Madhusmita Das^1^, Diana David^1^, Ilse Horo^1^, Anouk Van Hooij^2^, Maria Tió Coma^2^, Annemieke Geluk^2^ & Sundeep Chaitanya Vedithi^3^

**Comparative Analysis of genes between BI -Zero NR samples and BI positive NR samples:**

Given the diverse Ridley Jopling (RJ) and bacteriological index (BI) classification across the NR samples, we did a differential gene expression analysis of the non-reactional samples that have BI of zero in comparison those that have positive BI. We noted that genes ML0247(Putative arsenate reductase), ML2269 (Putative hydrolase), ML2296 (Putative membrane protein) and ML1182(PPE-family protein) are over expressed in BI Zero NR samples whereas ML1466 (50S ribosomal protein L27) and ML1180 (Putative ESAT-6-like protein X) are upregulated in BI positive samples.


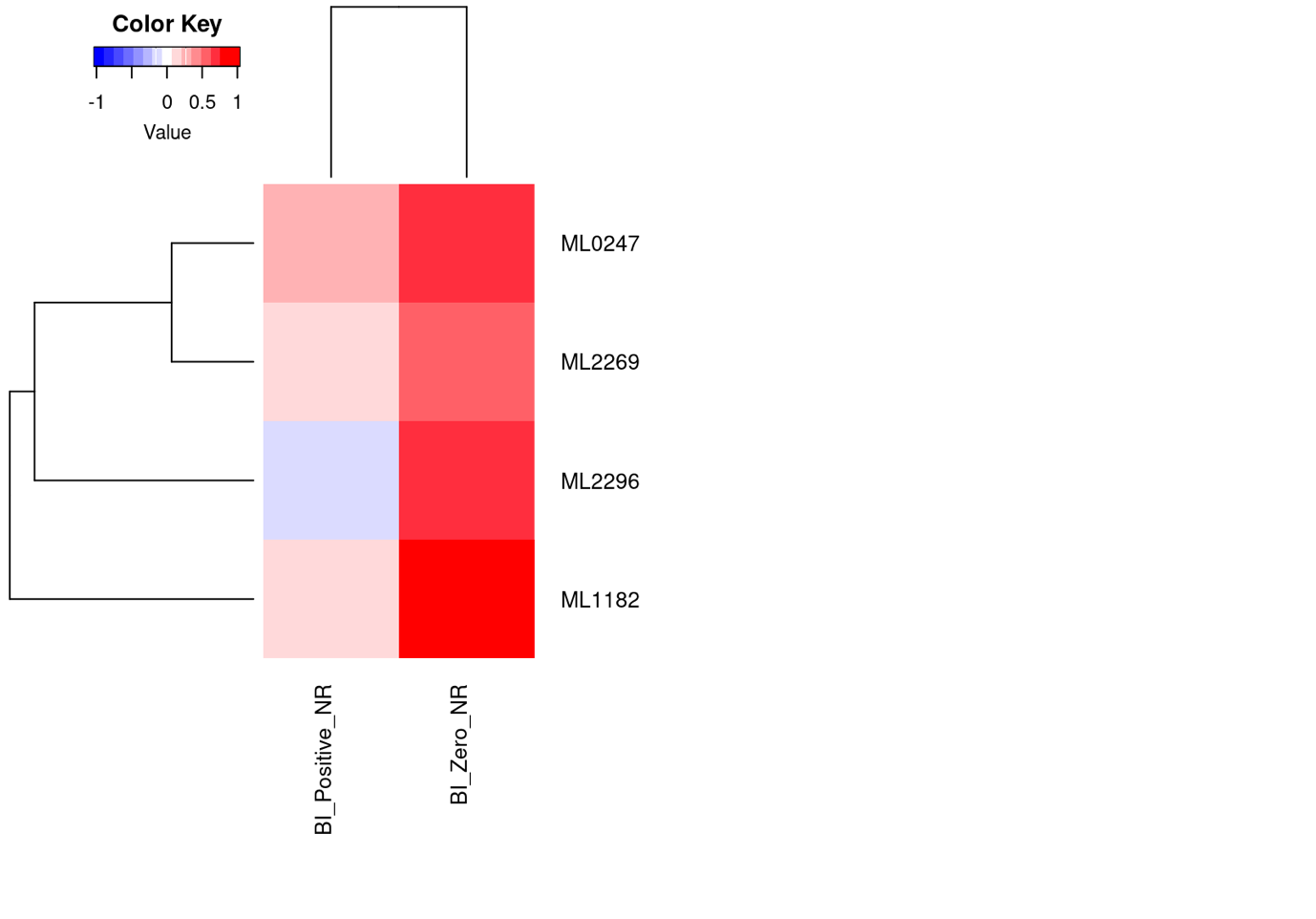
**Fig-S1** – Heatmap of differentially expressed genes in Non-Reactional (NR) samples whose bacteriological index (BI) is zero in comparison to NR samples with positive bacteriological index. A: Genes that are overexpressed in BI -Zero NR samples when compared to BI positive NR samples. B: Genes that are overexpressed in BI positive NR samples when compared to BI Zero NR set.


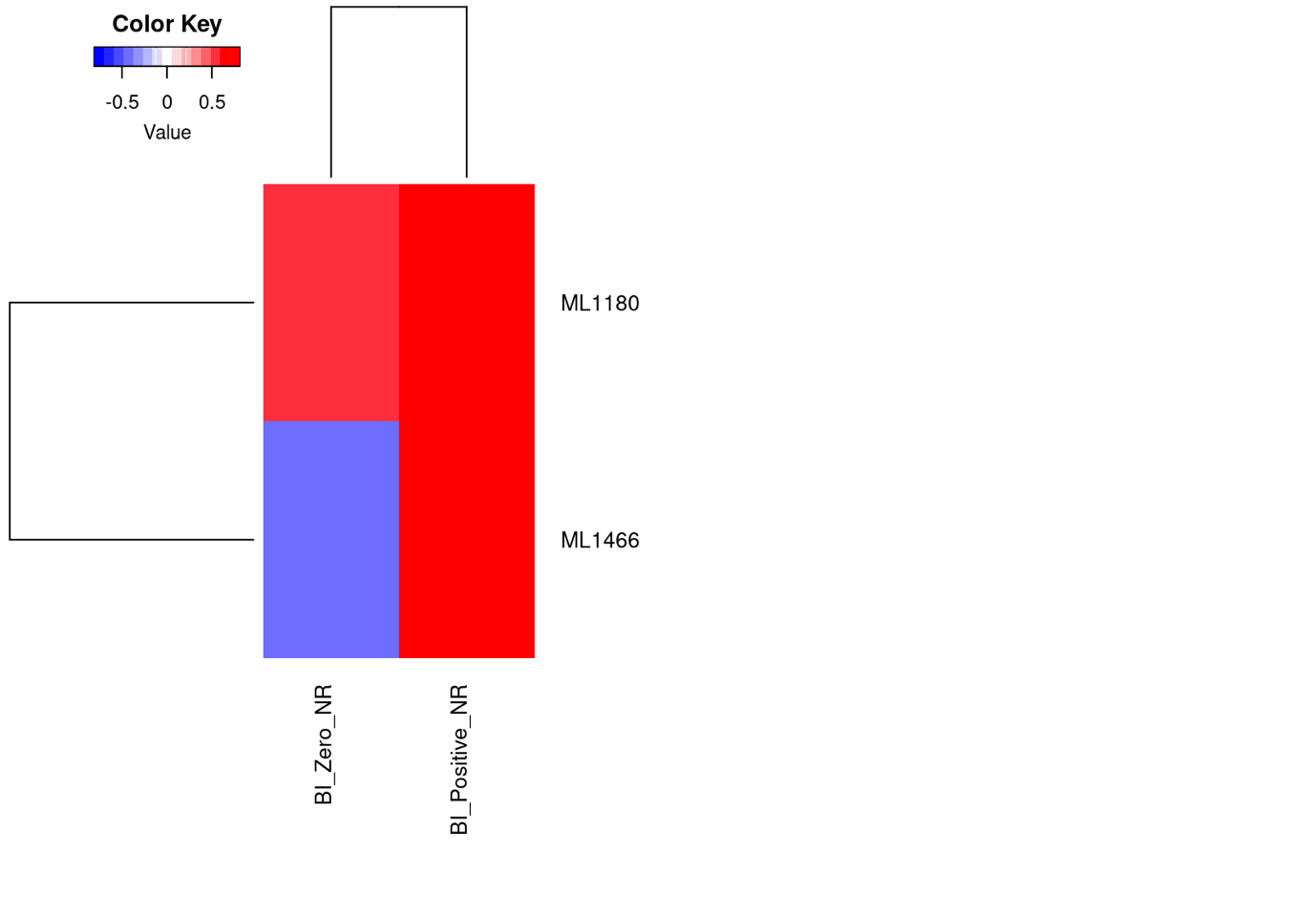
S1: A S1: B

**Fig-S2:** A: Heatmap of the DEGs that are upregulated in T1R when compared to NR leprosy cases with TT/BT – RJ Classification. B: DEGs that are upregulated in T2R when compared to NR leprosy cases with BL/LL Classification. From the analysis, the top10 differentially expressed genes remained the same before and after splitting the NR samples based on RJ classification.


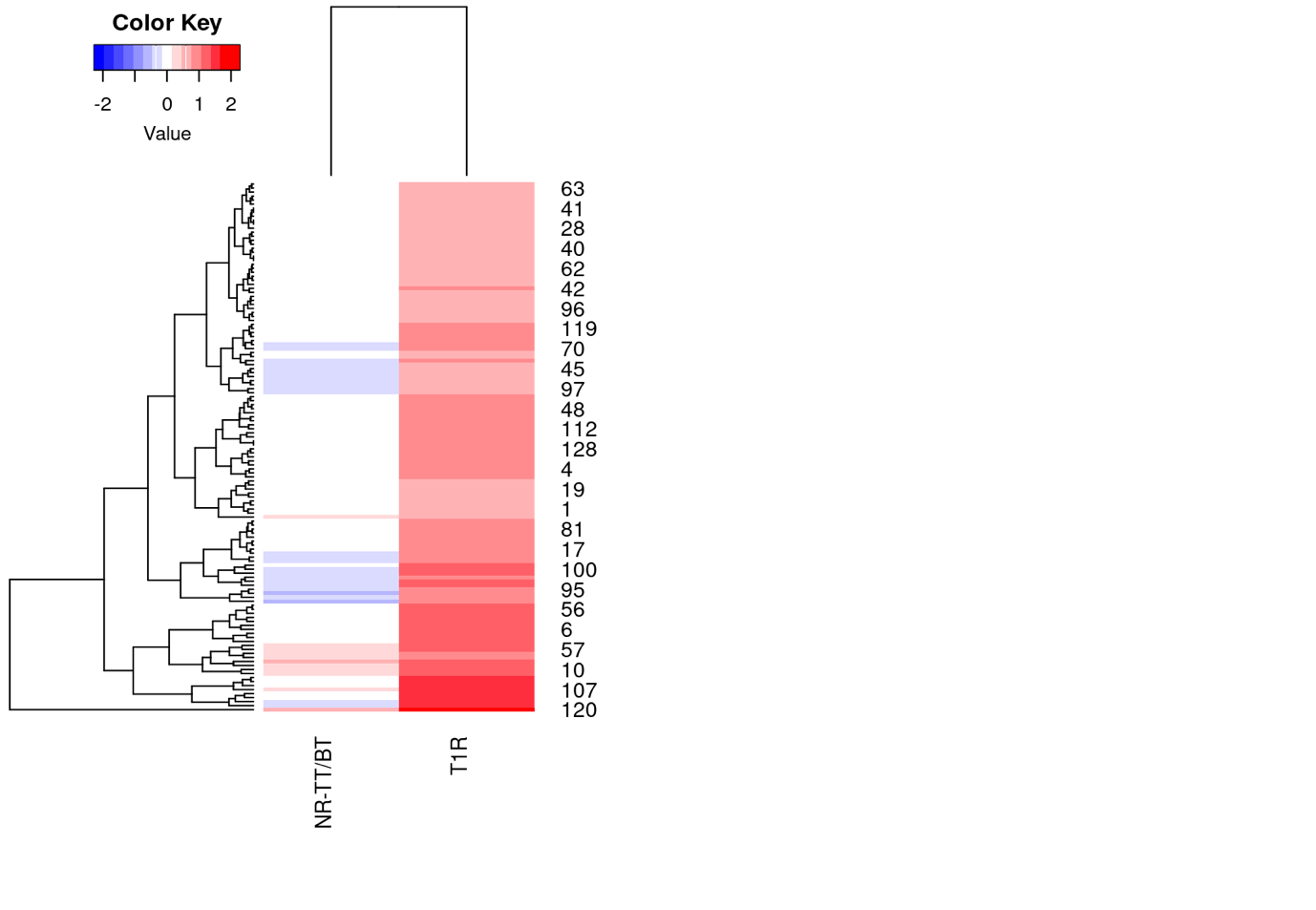
S2: A


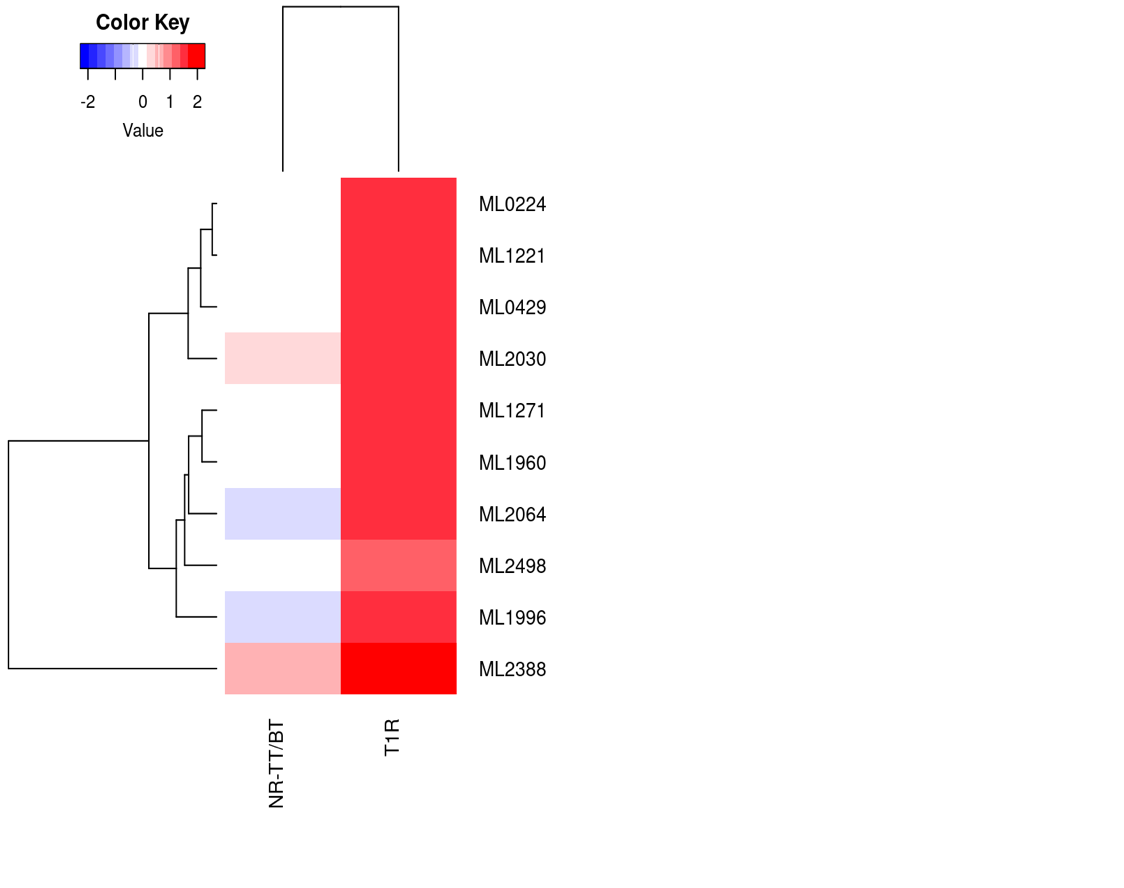


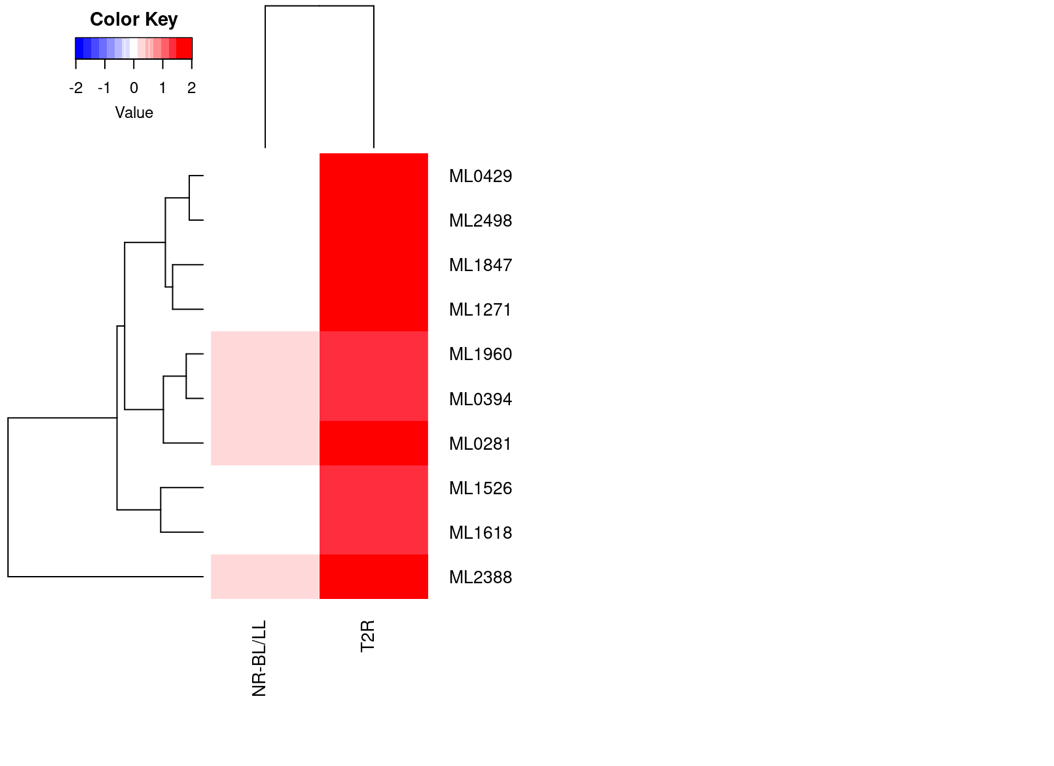

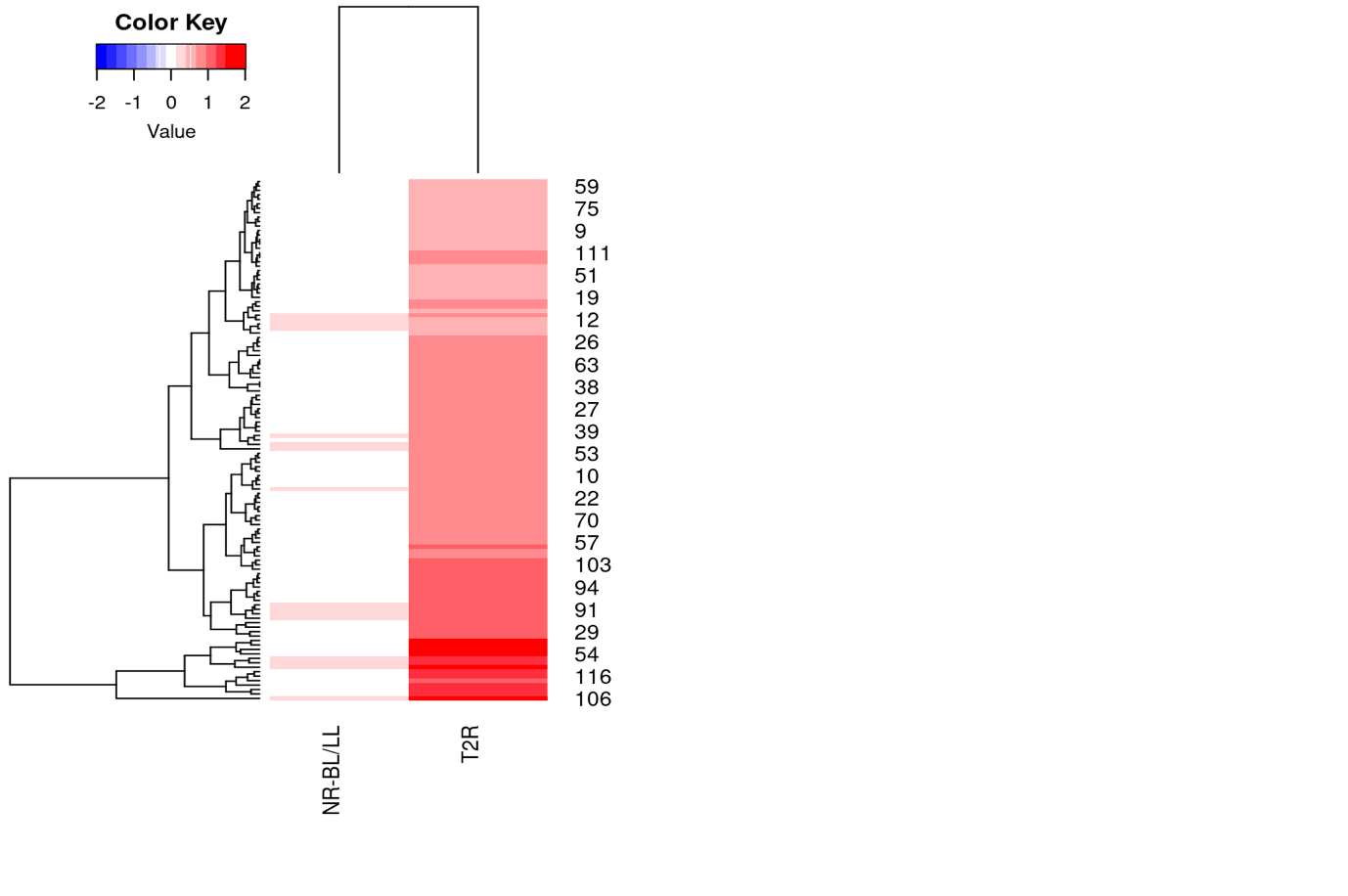
S2: B

**Supplementary Table-S3:** List of ThermoFisher Gene Expression Assays for human immune genes.

| Gene | ThermoFisher Gene Expression Assay Code |
| --- | --- |
| IL10 | Hs00961622_m1 |
| FCGR1B | Hs02340031_m1 |
| CD8A | Hs00233520_m1 |
| CTLA4 | Hs00175480_m1 |
| GNLY | Hs01120098_g1 |
| PRF1 | Hs00169473_m1 |
| CCL2 | Hs00234140_m1 |
| OAS1 | Hs00973635_m1 |
| IFI44 | Hs00197427_m1 |
| IFI6 | Hs00242571_m1 |
| CXL10 | Hs00171042_m1 |
| GAPDH | Hs02786624_g1 |
